# Chlorogenic acid compounds from sweetpotato (*Ipomoea batatas* L.) leaves facilitate megakaryocyte differentiation and thrombocytopoiesis *via* PI3K/AKT pathway

**DOI:** 10.3389/fphar.2024.1414739

**Published:** 2024-08-21

**Authors:** Yi Zhang, Lu Zhao, Liping Wang, Ruixue Yue, Hong Zhu, Wenting Zhang, Jian Sun, Zifeng Zhang, Daifu Ma

**Affiliations:** ^1^ Key Laboratory for Biotechnology on Medicinal Plants of Jiangsu Province, School of Life Science, Jiangsu Normal University, Xuzhou, China; ^2^ Xuzhou Institute of Agricultural Sciences in Jiangsu Xuhuai District, Xuzhou, China; ^3^ Jiangsu Key Laboratory of New Drug Research and Clinical Pharmacy, School of Pharmacy, Xuzhou Medical University, Xuzhou, China

**Keywords:** sweetpotato leaves, chlorogenic acid compounds, megakaryocyte, idiopathic thrombocytopenic purpura, PI3K/Akt pathway

## Abstract

Idiopathic thrombocytopenic purpura (ITP) is an autoimmune disorder characterized by antiplatelet autoantibodies, thrombocytopenia, and bleeding, however, its treatment options are limited. In this study, a kind of active component, chlorogenic acid compounds (CGAs) from sweetpotato leaves was extracted out to explore its medicinal value and provide novel therapeutic strategies for the treatment of ITP. CGAs was isolated by ionic liquids-ultrasound (IL-UAE), which contains six isomers of chlorogenic acid with total purity of 95.69%. The thrombopoietic effect and mechanism of CGAs were investigated using *in silico* prediction and experimental validation. The changes of HEL cells morphology in volume and the increase in the total cell percentage of polyploid cells indicated that CGAs could promote megakaryocyte differentiation. Meanwhile, CGAs could promote platelet formation in a murine model of ITP, which was established by injection of antiplatelet antibody. Further quantitative proteomics analysis and Western blot verification revealed that CGAs could activate PI3K/AKT signaling pathway, which confirmed the mechanism prediction. It suggested that CGAs may provide a novel therapeutic strategy that relies on the PI3K/AKT pathway to facilitate megakaryocyte differentiation and platelet production.

## 1 Introduction

Idiopathic thrombocytopenic purpura (ITP) is a prevalent hematologic disorder marked by destructive thrombocytopenia, significantly impacting patients’ quality of life (Wen et al., 2022). Platelets, derived from the cytoplasm of mature megakaryocytes in the bone marrow, play a pivotal role in hemostasis, thrombosis, and immunoregulation (Zhou et al., 2022).

Patients with severe bleeding in ITP are managed with platelet transfusions and intravenous immune globulin (Neunert et al., 2011). While platelet transfusions provide short-term relief, intravenous immune globulin, though costly, demonstrates significant efficacy and is not typically used as a first-line treatment option (Godeau et al., 2002). In patients without severe bleeding, treatment decisions are influenced by the platelet count and other clinical factors (Cooper and Ghanima, 2019). Pharmacological regimens remain the preferred therapeutic option for managing ITP. Nevertheless, it is important to note that the prolonged use of glucocorticoids, a common treatment for ITP, may lead to significant long-term side effects (Mithoowani et al., 2016). The therapeutic efficacy of thrombopoietin-receptor agonists in treating ITP may not align with expected outcomes due to associated side effects, while the mechanism of unresponsiveness to these agents remains unresolved (Chakraborty et al., 2020). The emergence of monoclonal antibodies such as rituximab has ushered in a new era in autoimmune disease treatment9. However, the high cost and uncertain long-term effectiveness present challenges. As a result, there remains a shortage of definitive curative drugs for ITP, highlighting the urgent need for ongoing exploration of new promising compounds.

Natural products have historically served as a fundamental source for the discovery and development of new drugs, owing to their often potent biological activity. Polyphenols, as secondary plant compounds, have attracted considerable attention due to their diverse biological functions. Structurally characterized by benzene derivatives with multiple hydroxyl substitutions, polyphenols are classified into distinct groups including flavonoids, lignans, tannins, astragalosides, ellagic acid homologs, gallic acid homologs, and curcumin (Croft, 2016; Rothwell et al., 2017). Chlorogenic acid compounds (CGAs), derived from caffeic acid and quinic acid, exhibit a range of biological functions, including antioxidant, antibacterial, anti-inflammatory, and neuroprotective activities. They show promise as physiologically active substances with beneficial effects on human health, supported by various epidemiological, biological, and biochemical data (Zhu et al., 2022).

Sweetpotato (*Ipomoea batatas* L.) is a high-yielding crop, with both its tubers and leaves being edible. The health-promoting biological activities in sweetpotato leaves are primarily attributed to phenolic acids and flavonoids (Wang, Nie and Zhu, 2016). Previous studies have highlighted key constituents in sweetpotato leaves, such as caffeic acid and monocaffeoylquinic acid, known for their potent antioxidant properties (Sun, Mu, Song, Ma and Mu, 2018). Despite being a rich source of chlorogenic acids (CGAs), sweetpotato leaves are currently underutilized, with only a small fraction of this valuable resource being consumed as a fresh vegetable. Therefore, there is a need for further exploration of the CGAs present in sweet potato eaves to uncover their potential pharmacological benefits.

In the present study, we extracted CGAs from sweetpotato leaves by IL-UAE and probed the molecular mechanism of CGAs action on ITP *via* a network pharmacology approach. We investigated the effects of CGAs on megakaryopoiesis using HEL cells and evaluate the effects of CGAs on thrombopoiesis using antiplatelet antibody-induced ITP mice. Meanwhile, quantitative proteomics and Western blotting analyses confirmed that the potential mechanisms of CGAs are related to activating the PI3K/AKT signaling pathway. In summary, this study contributes significant insights by integrating a combined computational analysis with experimental trials to investigate the thrombopoietic effects and underlying mechanisms of CGAs in the potential treatment of ITP.

## 2 Materials and methods

### 2.1 Materials

**FIGURE 1:**
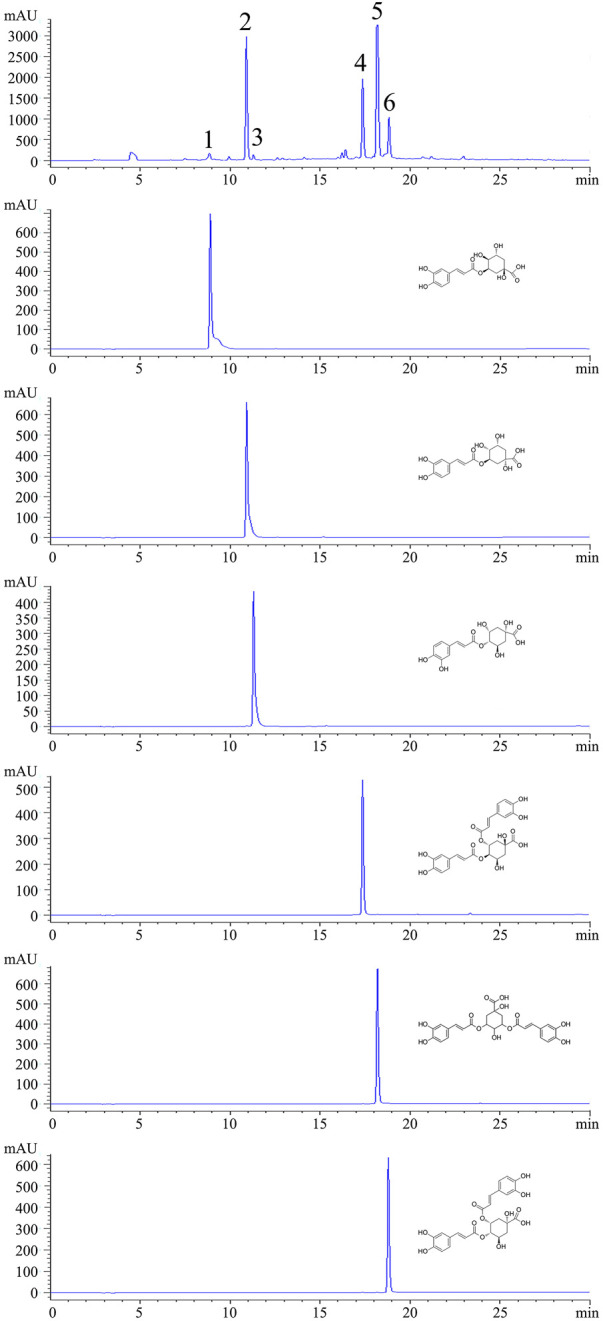
HPLC chromatograms of CGAs from sweetpotato and individual standards. (Peak1: 5-CQA. Peak2: 3-CQA. Peak3: 4-CQA. Peak4: 3,4-CQA. Peak5: 3,5-CQA. Peak6: 4,5-CQA.)

Chlorogenic acid (3-CQA), Cryptochlorogenic acid (4-CQA), Neochlorogenic acid (5-CQA), Isochlorogenic acid A (3,5-CQA), Isochlorogenic acid B (3,4-CQA) and Isochlorogenic acid C (4,5-CQA) were purchased from Chengdu Push Bio-technology Co., Ltd. (Chengdu, China), and their structures are shown in Figure 1. 1-butyl-3-methylimidazolium nitrate ([BMIM]NO_3_) was gained from Chengjie Chemical Reagents Co., Ltd (Shanghai, China). Acetonitrile and formic acid were obtained from Mallinkrodt Baker (NJ, United States), and μLtra-pure water was acquired by an Milli-Q water purification system (MA, United States). All the other reagents were purchased from Sinopharm which were AR Grade (Beijing, China). HEL cells were purchased from the Chinese Academy of Sciences cell bank (Shanghai, China). RPMI 1640 medium and cell culture materials were obtained from Life Technologies-Invitrogen (CA, United States). FITC-CD41^−^and PE-CD42b were obtained from Biolegend (CA, United States) and Annexin V-FITC/PI apoptosis kit was purchased from BestBio (Shanghai, China). ELISA kits of IL-6 (AYE-37846), TNF-α (AYE-3565), M-CSF (AYE-2237), TPO (AYE-29938), IL-2 (AYE-39484), IL-10 (AYE-34262), TGF-β (AYE-2984) were purchased from Xinle (Shanghai, China). Giemsa solution and phalloidin staining were purchased from Solarbio (Beijing, China). Commercial antibodies were purchased from Cell Signaling Technology (MA, United States). Sweetpotatoes (cultivar ‘Simon No. 1’) were planted with standard production practice at the Xuzhou Institute of Agricultural Sciences in Jiangsu Xuhuai District (117.29°E, 34.28°N), and its leaves were harvested at the beginning of July 2022. Guinea pig and BALB/c mice were purchased from Changzhou Cavens Experimental Animal Co., Ltd. (Changzhou, China). All experimental and euthanasia procedures performed in this study were approved by the Institutional Animal Care and Use Committee of Xuzhou Medical University protocols (IACUC: 202209S020). The mice were housed in a room under the conditions of constant temperature (23°C ± 2°C) and humidity (60%), and a 12 h light/dark schedule (lights on 8:00–20:00), and the mice were given free access to food and water.

### 2.2 Production CGAs from sweetpotato leaves by IL-UAE

A 0.3 g aliquot of powders was added to a centrifuge tube containing 3 mL of [BMIM]NO_3_. After sonication for 1 h, the supernatant extract was obtained by centrifugation at 2000 rpm for 5 min. Subsequently, a 200 mL aliquot of extract was combined with an equal volume of methanol and vortexed. After centrifuged at 10,000 rpm for 10 min, the supernatant was obtained and filtered through a 0.22 μm membrane for LC-MS analysis.

### 2.3 Qualitative and quantitative analysis

Qualitative analysis using HPLC (Ji et al., 2018). HPLC analysis was conducted using an Agilent 1260 HPLC system, which includes a quaternary pump, a diode array detector, an autosampler, and a column compartment (Agilent, CA, United States). Samples were separated on an Eclipse XDB-C18 column (4.6 × 250 mm, 5 μm), with a flow rate of 1.0 mL/min and an injection volume of 2 μL. The mobile phase consisted of acetonitrile and water (0.1% formic acid), and a gradient elution program was employed as follows: 15:85 V/V at 0 min, 55:45 V/V at 25 min, 95:5 V/V at 30 min, and 95:5 V/V at 35 min.

Quantitative analysis using UPLC-QE-Orbitrap-MS(Zhuang et al., 2020). Briefly, 50 mg of frozen CGAs samples were extracted with 70% aqueous methanol. The samples were subsequently homogenized employing a blender, subjected to ultrasonication for 30 min, and centrifuged at 12,000 rpm for 10 min. Following this preparation, the sample solutions were scrutinized utilizing an UPLC-Orbitrap-MS system comprising a Vanquish UPLC and Q Exactive MS from Thermo-Fisher Scientific located in Waltham, MA, United States. The analytical conditions were as follows, UPLC: Column, Waters ACQUITY UPLC^®^ HSS T3 (1.8 μm, 2.1 mm × 50 mm). Flow rate, 0.3 mL/min; Injection volume, 2 μL. Solvent system, acetonitrile (0.1% acetic acid): water (0.1% acetic acid). Gradient elution program, 3:97 V/V at 0 min, 3:97 V/V at 1.0 min, 50:50 V/V at 5.0 min, 90:10 V/V at 6.0 min, 90:10 V/V at 7.0 min, 3:97 V/V at 7.1 min, 3:97 V/V at 9.0 min.

HRMS data were recorded on a Q Exactive hybrid Q-Orbitrap mass spectrometer equipped with a heated ESI source utilizing the SIM-MS acquisition methods. The ESI source parameters were configured as follows: Spray voltage at −2.8 kV, sheath gas pressure at 40 arb, auxiliary gas pressure at 10 arb, sweep gas pressure at 0 arb, capillary temperature at 320°C, and auxiliary gas heater temperature at 350°C. Data collection was executed on the Q-Exactive instrument utilizing Xcalibur 4.1 software and subsequently processed using TraceFinder™4.1 Clinical. The quantified data were exported in excel format for further analysis.

### 2.4 Network pharmacology

The main targets of the CGAs were predicted by network pharmacology. The compound-related targets were acquired from the PharmMapper platform based on the structural formula of chlorogenic acid isomers (Forouzesh, Samadi Foroushani, Forouzesh, et al., 2019). The gene targets associated with the activity of CGAs were identified as “drug targets” through target data. ITP targets were obtained as “disease targets” from the GeneCards, OMIM and DisGeNet databases after duplicate removal (Piñero et al., 2017). Ultimately, the mapping of “drug targets” onto “disease targets” led to the identification of the shared targets involved in compound-target disease relationships.

To appraise the interactions of the targets in a Protein-Protein Interaction (PPI) network, the information pertaining to the shared targets was imported into the String 12.0 database, with parameters set for multiple proteins and *Homo sapiens*. The PPI file was subsequently downloaded for further analysis (Szklarczyk et al., 2017). The PPI data was then imported into Cytoscape 3.10.1 software to optimize visibility and conduct topology analysis of the PPI network. Gene Ontology (GO) term and Kyoto Encyclopedia of Genes and Genomes (KEGG) pathway enrichment analyses were performed to decipher the functions of differentially expressed genes, which were also used to predict the potential mechanism of drug action.

We entered the gene symbols of the common targets into the Database for Annotation, Visualization and Integrated Discovery, a database with integrated GO and KEGG modules (H. d. W, Huang et al., 2009). The GO analysis reflected a three-category evaluation of CGAs action, including the CGAs influenced genes in the biological process (BP), molecular function (MF), and cellular component (CC) categories. The KEGG analysis was performed for target-protein-related pathway screening to discover the key mechanism underlying CGAs driven recovery from ITP. The results obtained from the pathway enrichment analyses were prepared for visualization as a bubble diagram using the OmicShare platform.

### 2.5 Cell culture

HEL cells were grown in complete RPMI 1640 medium containing 10% foetal bovine serum (Gibco, MA, United States), 100 U/mL penicillin, and 100 mg/mL streptomycin (Beyotime, Nanjing, China) at 37°C in a humidified incubator with a 5% CO_2_ atmosphere.

### 2.6 Measurement of megakaryocyte differentiation

HEL cells were seeded into 6-well flat-bottomed plates at an initial cell density of 4.0 × 10^4^ cells/well. The cells were treated with 0.2 mg/mL and 0.1 mg/mL of CGAs to induce their differentiation into megakaryocytes. The culture medium was refreshed every 2 days. As an indicator of megakaryocyte differentiation, an increase in cell size was monitored (Limb et al., 2015; Huang et al., 2016). On the 8th day of treatment with CGAs, differentiated megakaryocytes that exhibited a size increase of at least 1.5-fold compared to undifferentiated HEL cells.

Following an 8-day incubation period, Giemsa staining was employed to visualize cell morphology under a microscope (Stockert et al., 2014). The harvested cells were washed twice with phosphate-buffered saline before being stained with Giemsa solution (Solarbio, Beijing, China) for 15 min. Multiploid cells were observed and captured under a microscope at ×500 magnification.

HEL cells were harvested for F-actin staining by phalloidin staining (Solarbio, Beijing, China) according to the manufacturer’s instructions. Initially, the cells were fixed with 4% paraformaldehyde for 15 min and permeabilized with a 0.05% Triton X-100 solution for 10 min. Subsequently, after washing with phosphate-buffered saline, TRITC-conjugated phalloidin working solution was applied to the cells to allow for infiltration. The cells were then incubated in the dark at room temperature, followed by another round of washing with phosphate-buffered saline. DAPI was used to restain the cell nuclei. Finally, the stained cells were imaged under a fluorescence microscope at ×500 magnification.

### 2.7 Megakaryocytes ploidy assay

Following eight consecutive days of CGAs treatment, HEL cells were centrifuged and prepared for staining after removal of the supernatant. A mixture of DAPI staining solution and 4% paraformaldehyde fixative solution was prepared at a volumetric ratio of 1:100 for nuclear staining. The cells were then incubated in the dark with the staining solution for 15 min and subsequently analyzed using a high-content cell imaging analysis system (Molecular Devices, ImageXpress Micro 4, CA, United States).

### 2.8 Flow cytometry analysis

HEL cells, harvested 8 days post-CGAs intervention, were subjected to a series of procedures. Initially, the cells were washed once with ice-cold phosphate-buffered saline, followed by the addition of 3 μL of FITC-CD41 and PE-CD42b (Biolegend, CA, United States), and then incubated for 30 min at room temperature in the dark. Then the cells were resuspended in 400 mL of phosphate-buffered saline and analyzed using a flow cytometer. CD41 expression was assessed using the fluorescein isothiocyanate (FITC) channel, while CD42b level was examined using the PE channel. The quantitation of the test biomarker expressions was based on the percentage of CD41^+^/CD42b^+^ cells. In addition, flow cytometry with Annexin V-FITC/PI double staining was employed to detect the CGAs-induced apoptosis of HEL cells. Following treatment, cells were harvested for apoptosis detection in accordance with the instructions provided in the Annexin V-FITC/PI apoptosis kit (BB-4101-50T, Shanghai, China).

### 2.9 Preparation of antiplatelet antibodies

The antiplatelet antibodies were generated following previously published methods (Arnott, Horsewood and Kelton, 1987; Lin et al., 2015). In brief, platelet pellets from BALB/c mice were harvested and used to immunize guinea pigs *via* subcutaneous injection at two sites on the paw and abdomen, as well as intradermal injection at two sites on the back on days 7, 14, and 28 (100 mL for each site). The guinea pig sera containing antiplatelet antibodies were collected at day 35. We used saturated ammonium sulfate to precipitate and purify the antiplatelet antibodies. The purified antiplatelet antibodies were dialyzed with 0.01 mol/L phosphate-buffered saline (pH 7.4) in the dialysis bag at 4°C for 24 h.

### 2.10 Establishment of a ITP mouse model and treatment with CGAs

The ITP mouse model was established as previously described with minor modifications (Arnott et al., 1987; X et al., 2015). The mice were randomly divided into four groups: Control group, Model group, High-dose group and Low-dose group. A dose of 16 mg/kg of antiplatelet antibody was injected intraperitoneally on days 1, 3, 5, 7, and nine in all groups except the normal control mice. We administered CGAs intragastrically each day for 15 consecutive days at high and low and dosages of 1,000 and 500 mg/kg, respectively. Blood samples were collected from the tail vein, and the platelets were counted every other 2 days following antibody injections.

### 2.11 ELISA measurements

The contents of interleukin-6 (IL-6), tumor necrosis factor alpha (TNF-α), macrophage-stimulating factor (M-CSF), thrombopoietin (TPO), interleukin-2 (IL-2), interleukin-10 (IL-10) and transforming growth factor-β (TGF-β) in plasma were assessed using commercially available ELISA kits (Xinle, Shanghai, China), following the manufacturer’s instructions.

### 2.12 Quantitative proteomics analysis

4D label-free quantification (4D-LFQ) mass spectrometry were used to screen differentially expressed proteins in bone marrow of ITP mice (Penkert et al., 2021; Kramer et al., 2022). In summary, protein samples were extracted (5 per group) and quantified using the bicinchoninic acid (BCA) Protein Assay Kit (Pierce, Thermo, United States). Take protein samples 100 μg and add TEAB (Triethylammonium bicarbonate buffer), which the final concentration of TEAB is 100 mM. Subsequently, introduce tris (2-carboxyethyl) phosphine to reach a final concentration of 10 mM and incubate the mixture for 60 min at 37°C. Then, add iodoacetamide to attain a final concentration of 40 mM and allow the reaction to proceed for 40 min at room temperature under dark conditions. Add a specific ratio of pre-cooled acetone (acetone: sample v/v = 6:1) to each sample and allow it to settle for 4 h at −20°C. After centrifugation for 20 min at 10, 000 g, collect the sediment and dissolve it in 100 µL of 100 mM TEAB solution. Subsequently, digest the mixture with Trypsin overnight at 37°C, vacuum dry the peptides, and resuspend them in 0.1% TFA. Following this, desalt the samples using HLB and vacuum dry them. Finally, determine the peptide concentrations using a peptide quantification kit (Thermo, Cat.23275).

Loading buffer was added to each tube to prepare samples for mass spectrometry analysis, with a concentration of 0.25 μg/μL for each sample. Trypsin-digested peptides were analyzed by an EASY nLC-1200 system (Thermo, United States) coupled with a timsTOF Pro2 (Bruker, Germany) mass spectrometer at Majorbio Bio-Pharm Technology Co. Ltd (Shanghai, China). The samples were separated on a Ionopticks UPLC C18-reversed phase column (75 μm × 250 mm, 1.6 μm) with a flow rate of 500 nL/min and a mobile phase consisting of acetonitrile (0.1% formic acid) and water (0.1% formic acid). Peptides were separated using an ultrahigh performance liquid phase system and subjected to a capillary ion source, followed by analysis with the timsTOF Pro2 mass spectrometer, with the electrospray voltage set at 1.5 kV. Peptide parent ions and their corresponding secondary fragments were identified and analyzed utilizing high-resolution TOF mass spectrometry. The secondary MS scan range spanned from 100 to 1700 m/z. Data acquisition using the timsTOF Pro2 instrument was performed in parallel accumulation serial fragmentation mode, capturing the second MS stage (for parent ion charge states of 0–5) with the 10 PASEF method. A dynamic exclusion time of 24 s was used for the MS/MS scan. Data collection soft is Compass HyStar (Brucker, Germany). Data analysis was accomplished by using MaxQuant software (2.0.3.1).

### 2.13 Evaluation of megakaryocytic histopathology in bone marrow

The ITP mice were sacrificed by cervical dislocation after CGAs treatment for nine consecutive days, and the femurs were collected. One femur from each mouse was used for evaluation of the megakaryocytic histopathology. The femurs were fixed with picric acid for 24 h, decalcified with 10% hydrochloric acid for another 24 h, and dewatered by gradient alcohol, then processed by xylene, embedded in paraffin, sectioned, and stained with hematoxylin and eosin. Megakaryocytic histopathology was evaluated under a microscope at ×500 magnification. The numbers of megakaryocytes in three microscopy fields per slide were counted.

### 2.14 Western blotting

Meanwhile, another femur from each mouse was used for evaluation of protein expression in the bone marrow. Total proteins were extracted from the bone marrow utilizing RIPA buffer and quantified using a BCA assay kit. Subsequently, following electrophoretic separation, the proteins were transferred onto a nitrocellulose membrane (0.45 μm, Merck Millipore, MA, United States). After a 1-h blocking step, the membrane was subjected to overnight incubation at 4°C with the primary antibodies including p-PI3K p85 (Tyr458)/p55 (Tyr199) (17366S), PI3K p110α (4255S), p-AKT (Ser473) (4060S) and AKT (pan) (4685S), followed by a peroxidase-conjugated secondary antibody. GAPDH was used as an internal control. The protein bands were visualized and quantified utilizing the Odyssey Infrared Imaging System.

### 2.15 Statistical analysis

Experiments were performed at least in triplicate. The data are expressed as the mean ± standard deviation. Statistical analysis was carried out using the two-tailed Student’s t-test by SPSS software (version 22.0). Values of *p < 0.05, **p < 0.01, #*p* < 0.05 and ##*p* < 0.01 were regarded as statistically significant.

## 3 Results

### 3.1 Identification of CGAs from sweetpotato leaves

By using HPLC chromatography, the components of CGAs obtained through IL-UAE were inferred. HPLC chromatograms of CGAs and individual standard caffeoylquinic acids derivatives are shown in Figure 1. A total of six chlorogenic acid isomers were identified, including 3-CQA, 4-CQA, 5-CQA, 3,5-CQA, 3,4-CQA, 4,5-CQA. The quantitation of the CGAs using UPLC-Orbitrap-MS are shown in Table 1. The coefficient of the regression equation *R* ^2^ was >0.99, indicating that the concentration and peak area have a close linear relationship. The CGAs is a fine, dry powder that contains six chlorogenic acid isomers with a purity of 95.69% ± 0.94% DW. Among the six chlorogenic acid compounds, three di-caffeoylquinic acids were the major components, accounting for 64.62%, which was consistent with our previous results from the total phenolic acid content of sweetpotato leaves purified using AB-8 macroporous resin. The most abundant compound was 3,5-CQA (36.47% ± 0.98% DW), followed by 3,4-CQA (14.73% ± 0.40% DW) and 4,5-CQA (13.42% ± 0.36% DW), which was similar to the previously reported. The content of 3-CQA was 26.41% ± 0.71% DW, which was higher than 4-CQA (3.73% ± 0.10% DW), while 5-CQA content (0.93% ± 0.02%) was the lowest among the chlorogenic acid isomers.

**TABLE 1:** Quantitated CGAs from sweetpotato leaves. Results expressed as mean ± standard deviation (n = 3).

Peak No.	Retention time (min)	Identification	Abbreviation	Standard curve	*R* ^2^	Peak area	Content (%, DW)
1	3.23	Neochlorogenic acid	5-CQA	Y = 5.195 × 10^4^X	0.9997	115.10 ± 3.10	0.93 ± 0.02
2	3.71	Chlorogenic acid	3-CQA	Y = 3.613 × 10^4^X	0.9979	3274.32 ± 88.09	26.41 ± 0.71
3	3.79	Cryptochlorogenic acid	4-CQA	Y = 6.296 × 10^4^X	0.9998	462.95 ± 12.45	3.73 ± 0.10
4	4.69	Isochlorogenic acid B	3,4-CQA	Y = 3.801 × 10^4^X	0.9951	1826.19 ± 49.13	14.73 ± 0.40
5	4.78	Isochlorogenic acid A	3,5-CQA	Y = 4.089 × 10^4^X	0.9967	4522.53 ± 121.67	36.47 ± 0.98
6	4.87	Isochlorogenic acid C	4,5-CQA	Y = 4.904 × 10^4^X	0.9971	1,664.67 ± 44.78	13.42 ± 0.36
Total chlorogenic acid isomers							95.69 ± 0.94

### 3.2 Network pharmacology prediction of the targets and the underlying mechanisms of CGAs against ITP

**FIGURE 2:**
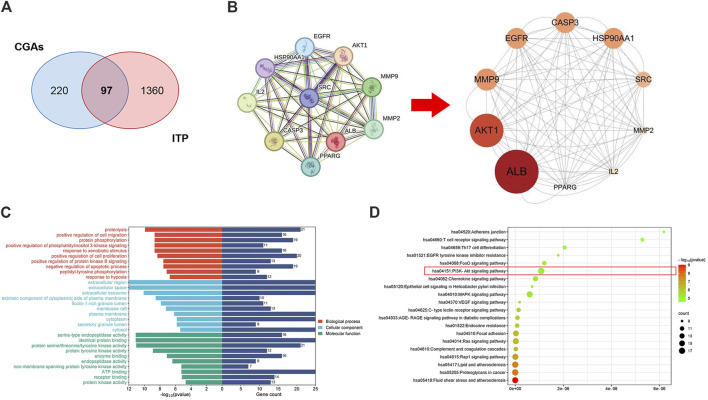
Network pharmacology prediction of CGAs activity in ITP treatment. **(A)** The Venn diagram of the shared targets of CGAs and in ITP. Blue: CGAs targets. Red: ITP targets. Pink: common targets. **(B)** PPI network based on the targets for CGAs in the treatment of ITP. **(C)** GO enrichment analysis of the hub targets for ITP treatment with CGAs. The 10 B P, 10 CC, and 10 MF terms with the greatest enrichment are listed in descending order of *p* values. **(D)** The 20 KEGG pathways enriched with hub targets for CGAs in treating ITP. The twenty most-enriched pathways were arranged in ascending order of *p* values. The bubble size indicates the number of enriched genes; its color signifies the corresponding *p*-value. The rich factor denotes the ratio of the target genes in a pathway to all the annotated genes in this pathway. The color of the bubble is related to the *p*-value.

The findings from network pharmacology revealed that PharmMapper identified 220 targets. Additionally, through the integration of data from three databases, a total of 1,360 targets associated with ITP were retained. Subsequently, 97 overlapping targets between CGAs and ITP were identified (Figure 2A). The common target genes of drugs and diseases were imported into String 12.0 database, parameter were set to the multiple proteins and *homo sapiens*, and the protein–protein interaction file was downloaded. A PPI network consisting of 10 nodes was constructed, and the core targets were determined using Cytoscape 3.10.1 (Figure 2B).

Thirty GO annotations were identified through enrichment analysis, comprising 10 terms each in the biological process (BP), cellular component (CC), and molecular function (MF) categories, as shown in Figure 2C. The BP terms primarily encompassed processes such as protein phosphorylation, positive regulation of phosphatidylinositol 3-kinase signaling, cell proliferation, and protein kinase B signaling. The CC terms were largely associated with extracellular regions, spaces, exosomes, and components of the cytoplasmic side of the plasma membrane. Meanwhile, the MF terms predominantly involved serine-type endopeptidase activity, identical protein binding, and various protein kinase activities. Additionally, the KEGG analysis identified 20 pathways potentially influenced by CGAs in regulating ITP, notably including Th17 cell differentiation, the PI3K-AKT signaling pathway, MAPK signaling pathway, RAGE signaling pathway, and Chemokine signaling pathway, as delineated in Figure 2D. The molecular mechanism underlying CGAs’ action in ITP may be closely linked to the PI3K/AKT signaling pathway.

### 3.3 CGAs-induce megakaryocyte differentiation

**FIGURE 3:**
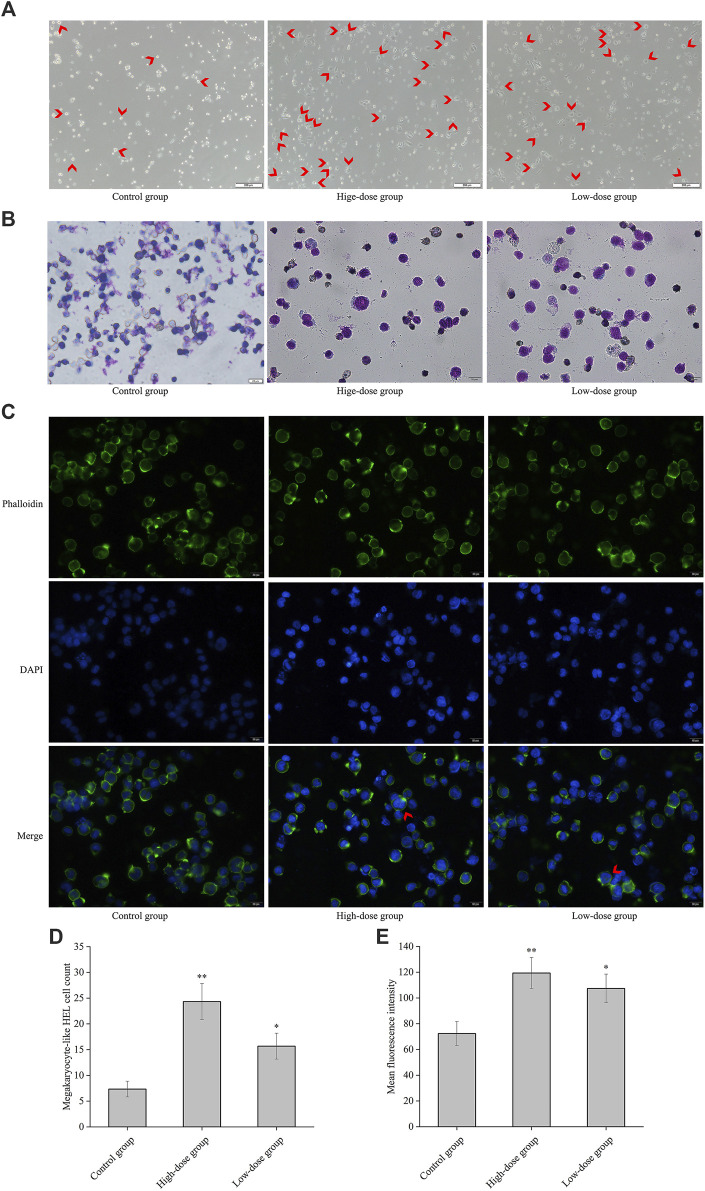
CGAs-induced HEL cell differentiation/polyploidization. **(A)** Differentiated megakaryocyte HEL cell count on the 8th day of treatment using a microscope at ×50magnification. The differentiated megakaryocyte HEL cells were significantly greater than that in the control cells after treatment with 0.2 mg/mL and 0.1 mg/mL CGAs. Arrows designate the salient region. **(B)** HEL cell nucleotype was observed with Giemsa staining under a microscope at ×200magnification. **(C)** F-actin cytoskeletons of HEL cells are shown with phalloidin staining 8 days after treatment with CGAs. Arrows designate the salient region. **(D)** The histogram displays the statistical result of differentiated megakaryocyte HEL cell count. *p < 0.05 and **p < 0.01, vs. control group. **(E)** The histograms display the mean fluorescence intensities as determined by statistical analysis. *p < 0.05 and **p < 0.01, vs. the control group.

As shown in Figure 3A, obvious differentiated megakaryocyte cells were observed. The cells treated with CGAs at 0.2 mg/mL and 0.1 mg/mL exhibited statistically significant differences (*p* < 0.01 or 0.05) in terms cytomorphology compared to the control cells (Figure 3D). Figure 3B demonstrates evident nuclear division in cells treated with CGAs on the 8th day, indicative of induced differentiation of HEL cells by CGAs. Figure 3C illustrates the dispersion of actin filaments, with arrows indicating actin bundles protruding from the cell membrane. Mean fluorescence intensities in the treated cells at both CGAS concentrations were significantly higher than those in the control cells (*p* < 0.01 or 0.05) (Figure 3E).

### 3.4 Regulatory effect of CGAs on the cell cycle and DNA ploidy in HEL cells

**FIGURE 4:**
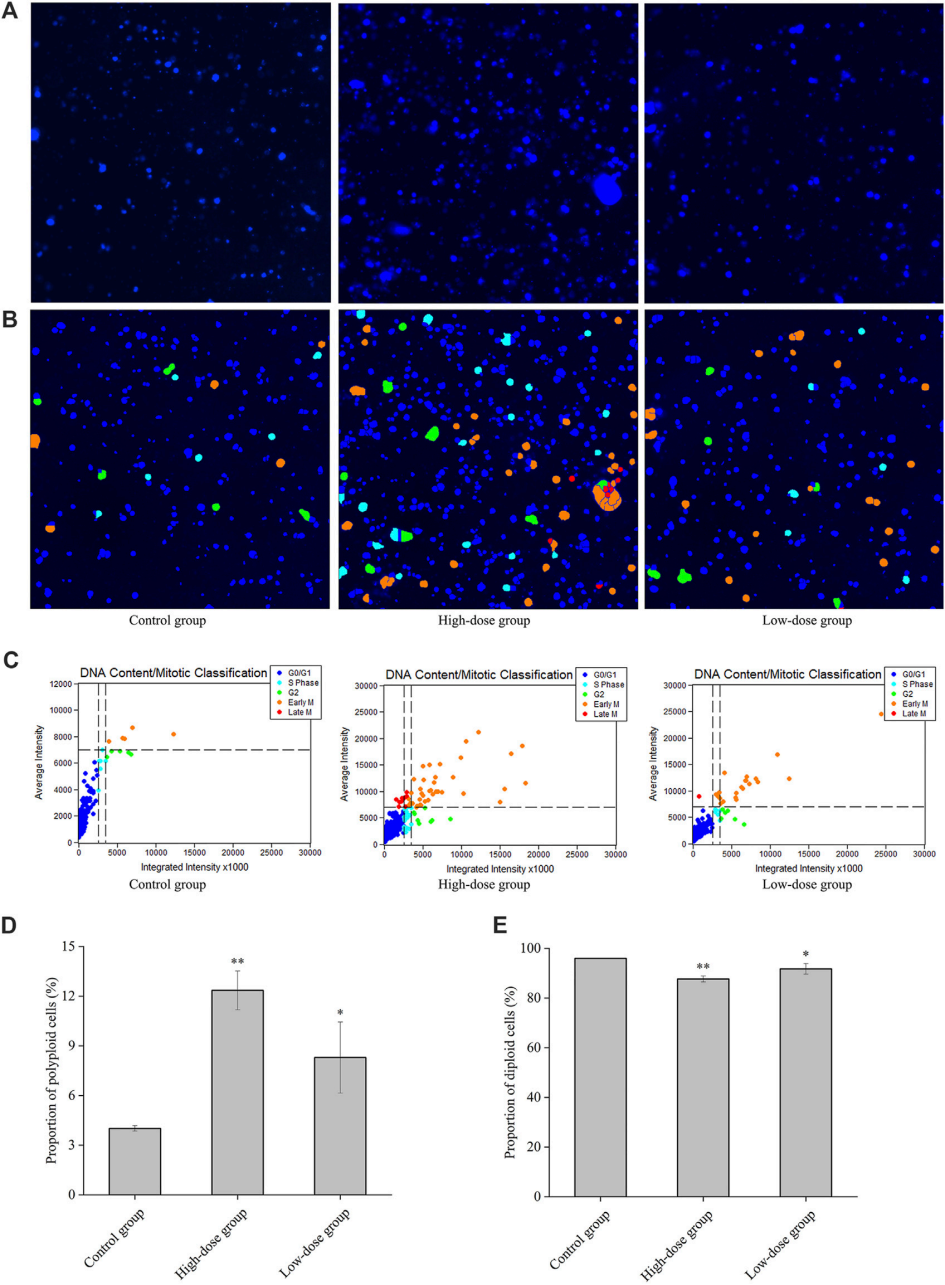
Regulatory effect of CGAs on the cell cycle and DNA ploidy in HEL cells. **A**: Fluorescence imaging by DAPI staining at ×200magnification. **(B, C)**: Cell cycle classification with DNA ploidy analysis. Blue, aquamarine blue, green, orange, and red cell labels represent the different phases of the cell cycle: G0/G1 (2 N), S (2 N), G2 (≥4 N), early M (≥4 N), and late M (2 N) phases, respectively. **(D)**: The proportion of polyploid cells. **(E)**: The proportion of diploid cells. *p < 0.05 and **p < 0.01, vs. the control group.

The polyploid proportion in these cells was subsequently detected with a high-content cell imaging analysis system, as shown in Figures 4A–C. The ploidy assay demonstrated that the proportion of polyploid cells significantly increased in a concentration-dependent manner 8 days post-CGAs intervention, compared with that in the control. However, the proportion of diploid cells decreased with increasing CGAs concentration (Figures 4D, E). These findings underscore the ability of CGAs to induce polyploidization in a dose-dependent manner, indicative of their role in promoting megakaryocyte differentiation.

### 3.5 The effect of CGAs on the CD41, CD42b and apoptosis levels in HEL cell

**FIGURE 5:**
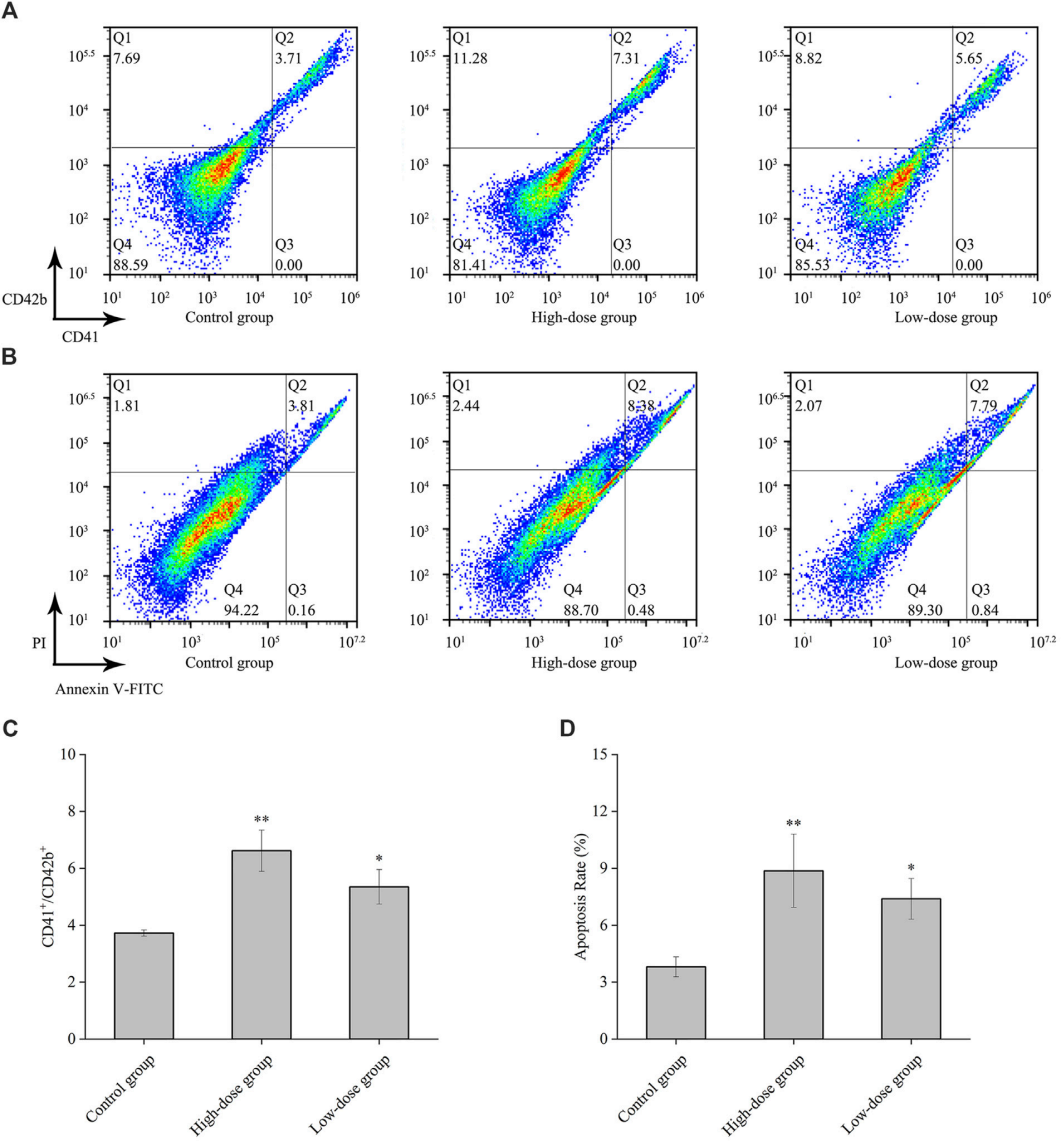
CD41, CD42b and apoptosis levels by CGAs in HEL cells. **(A)** The expression of CD41 and CD42b levels in HEL cells were measured on the 8th day after treatment with 0.2 mg/mL and 0.1 mg/mL CGAs. **(B)** Apoptotic cells of HEL cells treated CGAs concentrations were measured by flow cytometry using an Annexin V-FITC/PI apoptotic detecting kit. **(C)** The histograms show the statistical analysis of the cells percent with CD41^+^/CD42b^+^. **(D)** Quantitative analysis of apoptosis rate in different treatments. *p < 0.05 and **p < 0.01, vs. the control group.

Changes in the expression of characteristic molecular markers are commonly used to evaluate differentiation of megakaryocytes (Zhang et al., 2019). All platelets are the progeny of megakaryocytes, and megakaryocytes that produce functional platelets are marked as CD41^+^CD42b^+^ (Z. N et al., 2019). In this study, we analyzed the expression levels of cell surface CD41 and the megakaryocytic differentiation-associated antigen CD42b. The results demonstrated a significant enhancement in the population of CD41 and CD42b antibody-stained cells following CGA treatment (Figures 5A, C). This finding confirms the upregulation of CD41 and CD42b expression during the differentiation of megakaryocytes.

Proplatelet formation necessitates localized apoptosis to facilitate the cytoskeletal rearrangements essential for platelet shedding (De Botton et al., 2002). The results from flow cytometry assay using Annexin V/PI staining showed that CGAs induced excessive apoptosis in megakaryocytes on the 8th day (Figure 5B), with a concentration-dependent effect (Figure 5D). Presently, some scholars considered that megakaryocytes could shed platelets by promoting apoptosis (De Botton et al., 2002), while some others considered that it was necessary to inhibit the apoptosis of megakaryocytes in order to survive and proceed safely through the process of platelet shedding (Josefsson et al., 2011). Based on preliminary results, we speculate that CGAs had a protective effect on megakaryocytes during the early stages of intervention. However, as time progresses, megakaryocytes shed proplatelets through increased apoptosis.

### 3.6 Effects of CGAs on peripheral platelet counts and cytokine levels in murine ITP model

**FIGURE 6:**
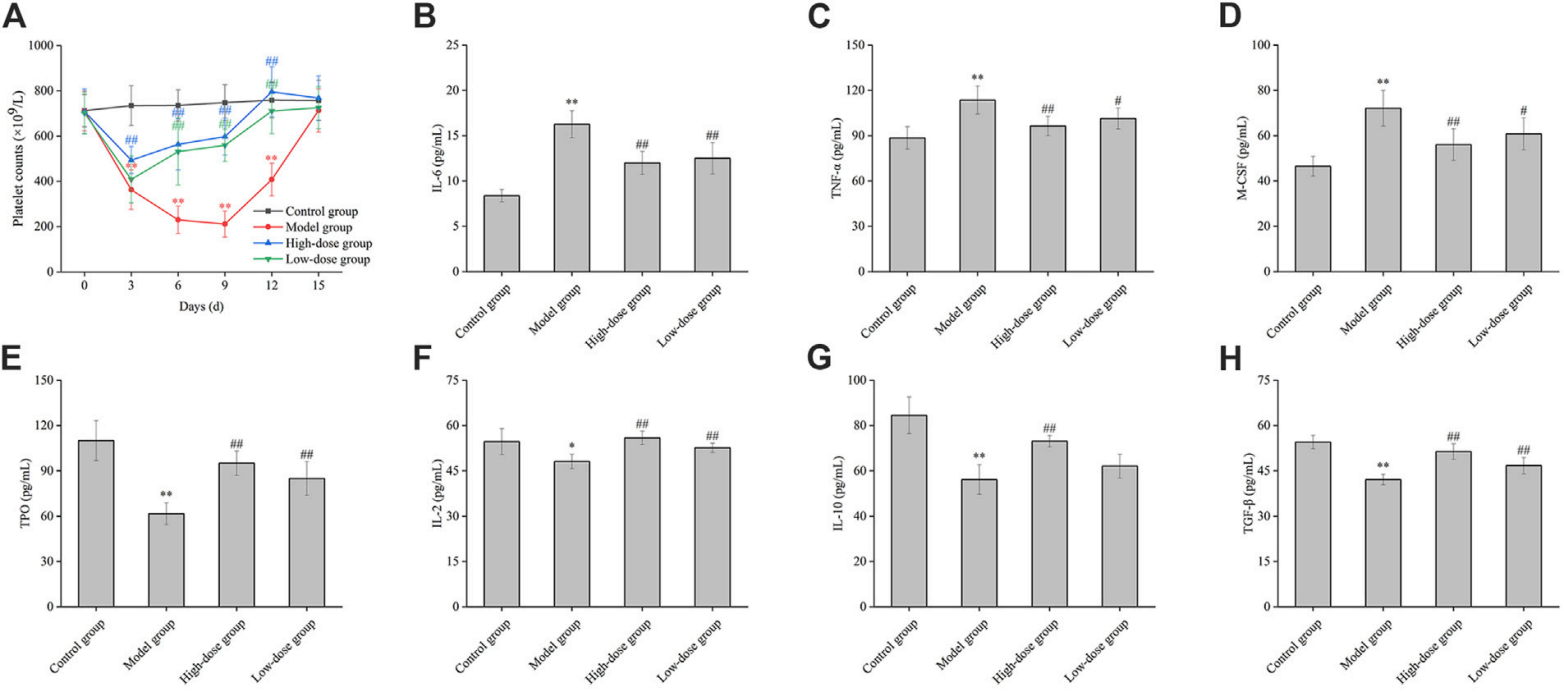
The effect of CGAs on the peripheral blood platelet counts, the contents of cytokine in ITP mice. **(A)**: ITP mice were established by intraperitoneal injection of antiplatelet antibody, which was derived from the sera of guinea pigs immunized with the platelets of BALB/c mice. ITP mice were treated with CGAs at high and low dosages (1,000 and 500 mg/kg) intragastrically administered daily for consecutive 15 days. Peripheral blood platelets were counted on days 0, 3, 6, 9, 12 and 15. **(B–H)**: Effect of CGAs on the level of IL-6, TNF-α, M-CSF, TPO, IL-2, IL-10, TGF-β. Plasma samples were collected at 9 days antiplatelet antibody post-injection, and the level in the culture media was evaluated using ELISA. *p < 0.05 and **p < 0.01 vs. the control group; #*p* < 0.05 and ##*p* < 0.01 vs. the model group.

Following the injection of antiplatelet antibodies, the peripheral blood platelet counts in the model group reached their nadir on day 9 (approximately 30% of normal levels) and gradually returned to normal by day 15 (Figure 6A). These observations indicate that the effect of high-dose CGAs began at the 3rd day (*p* < 0.01). The platelet levels of the high-dose group and low-dose group were significantly higher than the model group for 6,9 and 12 days (*p* < 0.01), indicating that CGAs could facilitate platelet recovery in ITP-afflicted mice.

As shown in Figure 6B, stimulation with antiplatelet autoantibodies markedly increased plasma IL-6 levels, an effect significantly attenuated by CGAs (*p* < 0.01). Furthermore, the reduced levels of TGF-β were significantly restored by both high and low-dose CGAs (*p* < 0.01) (Figure 6H). A statistically significant difference of IL-2 was observed between the model group and the normal group (*p* < 0.05), indicating a significant decrease in the detection value. Interestingly, the IL-2 concentration in the treatment groups demonstrated a dose-dependent increase with the administration of CGAs (Figure 6F). The difference of IL-10 between the model group and the normal group was statistically significant (*p* < 0.01). There were statistically significant differences between high-dose group and the normal group (*p* < 0.01). There was a significant increase of TNF-α and M-CSF in the model group compared with the control group (*p* < 0.01). Moreover, the concentration of TNF-α and M-CSF in high-dose group was significantly lower than that in the low-dose group (Figures 6C, D). Stimulation with antiplatelet autoantibodies notably reduced the level of TPO, and this degradation was significantly reversed by both high and low-dose CGAs (*p* < 0.01) (Figure 6E).

### 3.7 Effect of CGAs on the expression of proteins in bone marrow analyzed through proteomics

**FIGURE 7:**
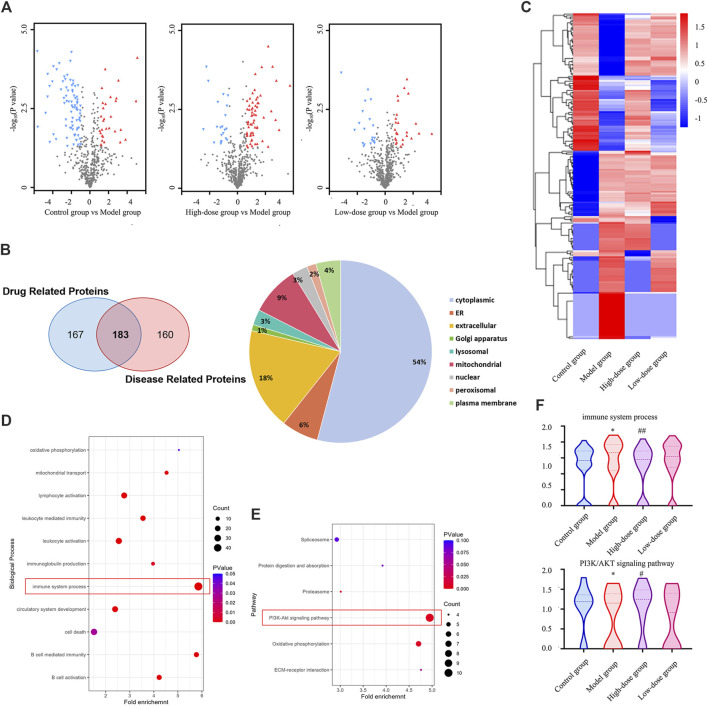
Proteomics analysis of differentially expressed proteins in the bone marrow of the ITP. **(A)** The volcano plot of the enriched differentially expressed proteins. **(B)** The Venn diagram of the shared targets of drug related proteins and disease related proteins. **(C)** The heatmap of the enriched differentially expressed proteins. **(D)** Enriched GO terms in terms of biological process. **(E)** Significantly enriched KEGG pathways based on the differentially expressed targets in bone marrow. **(F)** Effect of CGAs on immune system process and PI3K/AKT pathway. *p < 0.05 vs. the control group; #*p* < 0.05 and ##*p* < 0.01 vs. the model group.

To characterize the impact of CGAs on the bone marrow proteome in mice, tissue samples were subjected to proteomic analysis. Volcano plots of the differentially expressed proteins are shown in Figure 7A. Compared with the control group, the model group exhibited 120 upregulated and 223 downregulated proteins. Treatment with high-dose CGAs resulted in 246 upregulated and 104 downregulated proteins compared to the model group. Similarly, low-dose CGAs treatment led to 154 upregulated and 90 downregulated proteins relative to the model group. A heatmap (Figure 7C) was generated to depict the variations in protein expression across the control group, model group, and CGAs administration group. Subsequent analysis using a Venn diagram revealed 343 proteins exhibiting changes after disease onset, and 350 proteins showing alterations following high-dose administration. Through this, 183 key proteins, which are both implicated in the disease and responsive to pharmacological effects, were identified. Predominantly, the differential proteins are localized in the cytoplasm, with significant representation in extracellular, mitochondrial, and endoplasmic reticulum compartments as well (Figure 7B).

To delineate the functions of CGAs-regulated differentially expressed proteins, we conducted GO enrichment analysis (Figure 7D) and KEGG pathway enrichment analysis (Figure 7E). The GO enrichment analysis revealed that the differentially expressed proteins were enriched in various cell biological processes associated with the immune system, including B cell-mediated immunity and leukocyte-mediated immunity, suggesting a positive regulatory effect of CGAs administration on the immune system. KEGG pathway enrichment analysis proved that CGAs could significantly regulate PI3K/AKT signaling pathway, oxidative phosphorylation and ECM-receptor interactions, all of which are crucial for megakaryocyte differentiation and platelet production, and they may be associated with the potential molecular mechanism of CGAs treatment. The adjustment to the PI3K/AKT signalling pathway and immune system may be instrumental in facilitating ITP by CGAs (Figure 7F).

### 3.8 Effect of CGAs on the numbers of megakaryocyte and the protein expression of PI3K and AKT in bone marrow

**FIGURE 8:**
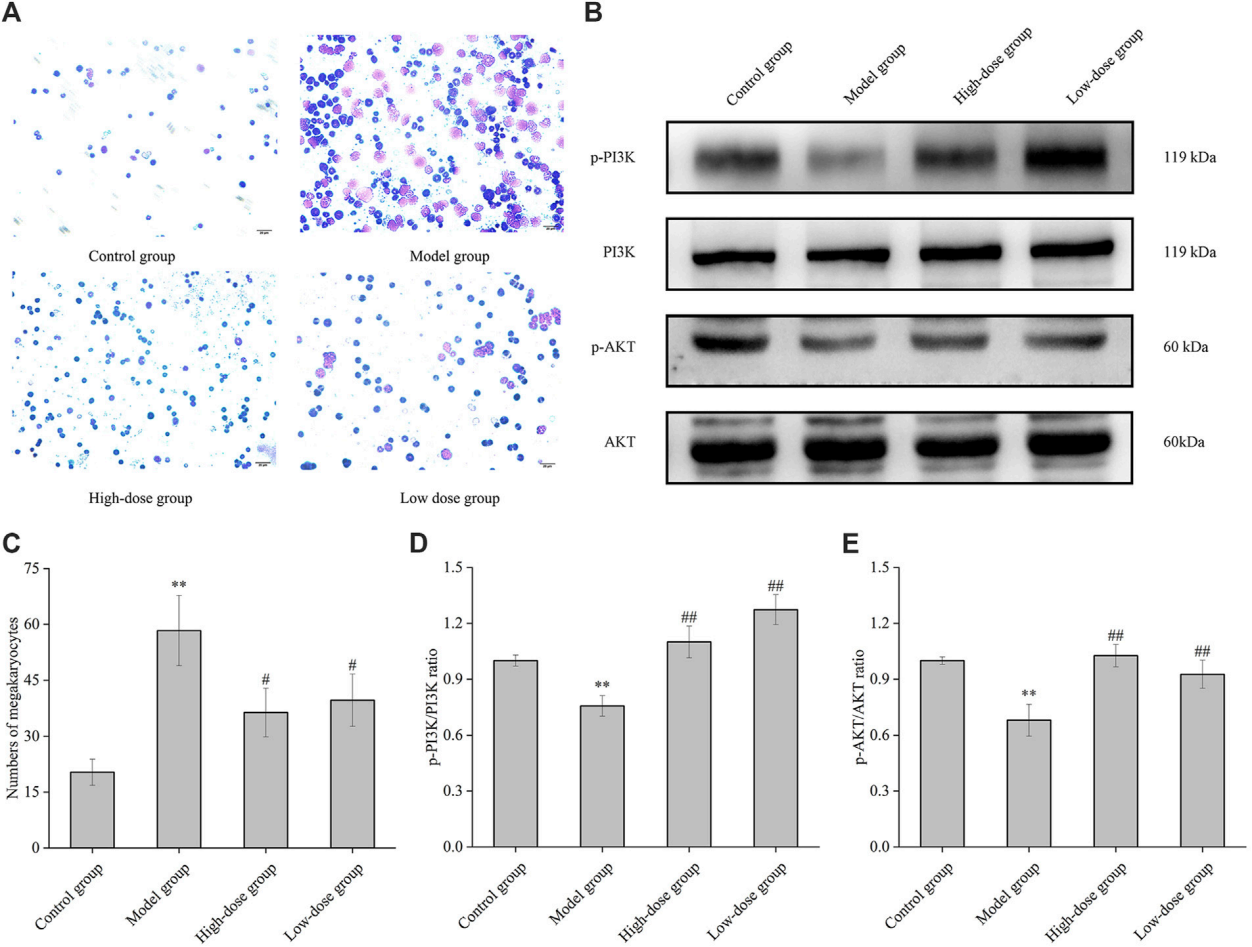
Megakaryocyte classical counting and PI3K and AKT proteins expression of mice bone marrow. **(A)** Bone marrow histopathology of normal group, model group, high-dose group (1,000 mg/kg of CGAs treatment) and low-dose group (500 mg/kg of CGAs treatment). **(B)** The p-PI3K, PI3K, p-AKT and AKT protein levels were determined by Western blotting. **(C)** Bar diagram of the numbers of megakaryocytes in the bone marrow. **(D, E)** The relative ratios of the protein levels were quantified. *p < 0.05 and **p < 0.01 vs. the control group; #*p* < 0.05 and ##*p* < 0.01 vs. the model group.

In the bone marrow of ITP mice, the count of megakaryocytes exhibited a significant increase compared to normal mice (*p* < 0.01) (Figure 8A). A bar diagram in Figure 8C illustrates the variations in megakaryocyte counts among normal mice, ITP mice, and those treated with high and low doses of CGAs. These results indicate that megakaryocyte levels were elevated in the bone marrow of ITP mice but decreased following CGAs treatment (*p* < 0.05). Notably, while megakaryocyte counts decreased post-CGAs treatment, indicating a partial reversal of compensatory increases, this phenomenon suggests a potential mechanism for CGAs in increasing peripheral platelet counts and effectively treating ITP in mice.

Phosphorylation levels of PI3K and AKT were significantly elevated in CGAs-treated ITP mice compared to those in the model mice (*p* < 0.05 or 0.01) (Figures 8B, D, E). These results underscore the capacity of CGAs stimulation to augment megakaryocyte differentiation and platelet production *via* the activation of the PI3K/AKT cascade.

## 4 Discussion

ITP is an autoimmune and inflammatory hematological disorder characterized by the immune-mediated destruction of platelets. The destruction of platelets in patients with ITP, mediated by the monocyte-macrophage system as a consequence of anti-platelet auto-antibody production, is intrinsically linked to immune dysregulation (Josefsson et al., 2011). In this condition, the decline in platelet count elevates the risk of hemorrhagic events. Clinically, individuals with ITP exhibit petechiae on the skin, alongside other hemorrhagic manifestations including epistaxis and gingival bleeding. Furthermore, purpura and ecchymosis may manifest on any area of the skin or mucosa, predominantly affecting the lower and distal upper extremities (Li et al., 2017). The primary treatment modalities for ITP, including corticosteroids and intravenous immunoglobulin. However, for corticosteroid dependent patients or those unresponsive to corticosteroids, therapeutic options include thrombopoietin receptor agonists, splenectomy, and rituximab (Baysal et al., 2021). Considering the side effects, cost, and efficacy of medications such as glucocorticoids and antibody drugs, natural remedies have significant advantages in treating ITP, a chronic disease.

Medications derived from natural sources are recognized as effective complementary approaches in managing chronic conditions such as ITP. These medications have shown significant efficacy in increasing platelet counts, reducing bleeding (Huang et al., 2013), and promoting the proliferation of granulocytes and megakaryocytes, thereby contributing to the overall management of the condition (Li et al., 2018). This study investigates the impact of CGAs from sweetpotato leaves on megakaryocyte differentiation and platelet production, encompassing both *in vitro* studies and *in vivo* experimentation. Th17 cells are now recognized for their pivotal role in numerous inflammatory and autoimmune diseases, roles previously attributed to Th1 lymphocytes. Conditions characterized by chronic inflammation and immune-mediated pathologies, such as rheumatoid arthritis, psoriasis, inflammatory bowel disease, and ITP, are closely linked to aberrant activation of Th17 cells. These cells produce cytokines such as IL-17, which promote the infiltration and activation of inflammatory cells, thereby initiating and perpetuating tissue inflammation. Elevated IL-17 levels observed in ITP patients suggest its contribution to immune dysregulation and heightened inflammatory responses, potentially influencing platelet reduction. Intervening to modulate the abnormal activation of Th17 cells presents significant therapeutic promise for managing ITP (M. G, Murdaca et al., 2011). Clinical studies have found that low oral doses of cyclosporin A in the treatment of systemic sclerosis can block the activation of nuclear factors such as NFAT and OAP. These nuclear factors are involved in the induction of mRNA transcription for various immune-activating and proinflammatory cytokines, thereby inspiring our exploration of the mechanisms of CGAs on ITP (Filaci et al., 2001). Chlorogenic acid, primarily found in green coffee and tea extracts, is a key dietary polyphenol with diverse therapeutic properties including antioxidant, antibacterial, hepatoprotective, cardioprotective, anti-inflammatory, neuroprotective, anti-obesity, antiviral, antimicrobial, and antihypertensive effects, alongside acting as a free radical scavenger and central nervous system stimulant (Naveed et al., 2018).

Sweetpotato leaves are excellent source of chlorogenic acids compared to other commercial vegetables (Islam et al., 2002). Our results carry important implications for the development of the medicinal value of sweetpotato leaves. Nevertheless, our study is constrained by the presence of multiple components within the compound, making it impractical to influence platelet generation and immune processes through various targets or signaling pathways.

## 5 Conclusion

Our research comprehensively outlines the impact of CGAs on megakaryocyte differentiation and platelet production, spanning from *in vitro* studies to *in vivo* experimentation, and from predictive modeling to experimental validation. Our results indicate that CGAs induce HEL cell differentiation, effectively enhance peripheral blood platelet counts in ITP mice, and modulate the PI3K/AKT signaling pathway in ITP management. Moreover, proteomics analysis suggests that CGAs hold promise as a dual-action treatment for ITP, promoting both megakaryocyte differentiation and proliferation while modulating immune function. Overall, CGAs present a promising avenue for ITP therapy, offering potential as a novel therapeutic agent.

## Data Availability

The data presented in the study are deposited in the ProteomeXchange Consortium, via the iProX partner repository, accession number PXD054945. Available at: https://www.iprox.cn//page/SCV017.html?query=PXD054945.

## Abbreviations

ITP: immune thrombocytopenia; CGAs: chlorogenic acid compounds; IL-UAE: ionic liquids-ultrasound [BMIM]NO3: 1-butyl-3-methylimidazolium nitrate; PPI: Protein-Protein Interaction; GO: Gene Ontology; KEGG: Kyoto Encyclopedia of Genes and Genomes; BP: biological process; MF: molecular function; CC: cellular component; FITC: fluorescein isothiocyanate; IL-6: interleukin-6; TNF-α: tumor necrosis factor alpha; M-CSF: macrophage-stimulating factor; TPO: thrombopoietin; IL-2: interleukin-2; IL-10: interleukin-10; TGF-β: transforming growth factor-β; 4D-LFQ: 4D label-free quantification; BCA: bicinchoninic acid.
